# Does implicit mentalizing involve the representation of others' mental state content? Examining domain-specificity with an adapted Joint Simon task

**DOI:** 10.1098/rsos.230239

**Published:** 2024-08-14

**Authors:** Malcolm K. Y. Wong, Marina Bazhydai, Calum Hartley, J. Jessica Wang

**Affiliations:** Department of Psychology, Lancaster University, Lancaster, UK

**Keywords:** implicit mentalizing, co-representation, joint action, domain specificity, Joint Simon effect

## Abstract

Implicit mentalizing involves the automatic awareness of others’ perspectives, but its domain-specificity is debated. The Joint Simon task demonstrates implicit mentalizing as a Joint Simon effect (JSE), proposed to stem from spontaneous action co-representation of a social partner's frame of reference in the Joint (but not Individual) task. However, evidence also shows that any sufficiently salient entity (not necessarily social) can induce the JSE. Here, we investigated the content of co-representation through a novel Joint Simon task where participants viewed a set of distinct images assigned to either themselves or their partner. Critically, a surprise image recognition task allowed us to identify partner-driven effects exclusive to the Joint task-sharing condition, versus the Individual condition. We did not observe a significant JSE, preventing us from drawing confident conclusions about the effect's domain-specificity. However, the recognition task results revealed that participants in the Joint task did not recognize their partner's stimuli more accurately than participants in the Individual task. This implies that participants were no more likely to encode content from their partner's perspective during the Joint task. Overall, this study pushes methodological boundaries regarding the elicitation of co-representation in the Joint Simon task and demonstrates the potential utility of a surprise recognition task.

## 1. Introduction

To mentalize is to understand that others have mental states, such as beliefs, knowledge, perspectives, etc. independent of one's own mental state [1]. This ability, also known as Theory of Mind [2], is crucial to an individual's navigation of the social world [3]. For instance, a chess or poker player may purposefully mentalize their opponent's emotions and intentions (e.g. whether the opponent is playing suspiciously, or possible actions which they may take) when deliberating the ideal game action [4]. According to the two-systems account of Theory of Mind [5,6], the ability to perform such a task is a form of *explicit mentalizing*: Sslow and effortful, but flexible and intentional mentalistic reasoning. This is in contrast with *implicit mentalizing*: rapid, spontaneous, and non-conscious *mentalizing* which does not involve linguistically mediated deliberation [7]. An example of implicit *mentalizing* may be found in a game of double table tennis, where a player may quickly and non-consciously read their partner's (and/or opponent's) movements, and automatically adjust their own movements correspondingly to facilitate (or inhibit) their attack [8]. Intriguingly, it has been found that implicit *mentalizing* occurred between individuals during shared tasks even when it was task-irrelevant and detrimental to task performance [9]. Such unintuitive findings have led to the exploration of the boundaries and underlying mechanisms of implicit *mentalizing*, with a particular focus on explaining how the apparent socially reactive nature of implicit *mentalizing* can be achieved spontaneously [10]. Within the contemporary literature, one heated point of debate lies in whether implicit *mentalizing* recruits *specific* cognitive modules dedicated exclusively to *mentalizing* in social situations (domain-specific accounts; e.g. [11,12]) or if it is driven by a *general* cognitive system, shared across both social and non-social situations (domain-general accounts; e.g. [13]). This question has prompted the usage of numerous experimental paradigms in an attempt to untangle the domain specificity of implicit *mentalizing*—however, a consensus has yet to be reached.

Of the myriad experimental paradigms used to investigate implicit mentalization, one of the most prominent tasks is the Joint Simon task [14]. The Joint Simon task was based on the seminal two-choice Simon task, which demonstrated that spatially defined responses (e.g. left versus right button-presses) to non-spatial stimuli features (e.g. discriminating between green versus blue stimuli) produced spatial compatibility effects (SCEs)—faster responses to spatially Compatible than Incompatible trials [15]. For example, in a standard two-choice Simon task (‘standard Simon task’ hereafter), participants may be told to press the left button whenever they saw blue stimuli, regardless of where the stimuli appear. Despite the fact that stimuli location (left versus right) is ostensibly task-irrelevant, it was found that participants were quicker and more accurate to respond to trials in which the blue stimuli appeared on the left of the screen (thus achieving stimulus-response compatibility) than the right [16].

The explanation generally accepted for this phenomenon is that the spatial overlap between the stimulus and response location assists in low-level response selection and that the discrepancy between the locations of the stimulus and the response button on incompatible trials requires disambiguation, even when those locations are irrelevant to the task at hand [17,18]. Indeed, SCE is attenuated when an individual undertakes half of the standard Simon task on their own (effectively an individual go/no-go task, as participants only respond to stimuli of one colour), as this removes the option for an alternate response location [19]. Of relevance to the domain specificity debate, SCE is crucially restored when two participants each undertake half of the Simon task side-by-side (also known as the Joint Simon task or the Joint go/no-go task), in which each participant is responsible for one button and one colour [14]. This is notable because each participant in the Joint Simon task is functionally performing the same task as a participant in the individual go/no-go task, and yet SCEs are only found in the Joint, but not Individual, Simon tasks—this set of results are known as the Joint Simon effect (JSE). According to a domain-specific account, the JSE demonstrates that participants in the Joint Simon task spontaneously represent their partner's task, including their visual perspective and frame of reference, even when detrimental to their own performance [20]. This action co-representation is thought to be a spontaneous, social response which inadvertently reinstates the spatial overlap between the stimulus and response location (as found in the standard Simon task), thereby resulting in the SCE [21,22].

Later studies building on the Joint Simon task provide further evidence for the apparently social nature of implicit *mentalizing*. It was found in multiple studies that socially related factors such as emotional valence can predict the JSE. For instance, when a (usually confederate) partner displays a bad mood [23], intimidation [24], or competitiveness [25], it was observed that the magnitudes of JSEs were attenuated, while opposite conditions in which the partners had a positive emotional valence elicited stronger JSEs. Relatedly, factors such as the participant's interpersonal closeness to their partner also influence the magnitude of JSEs [26]; for example, it has been reported that romantic partners evoked stronger JSEs in each other when compared to pairs of friends [27]. A similar line of results was found in comparisons between in-group and out-group paired performance in the Joint Simon task—the former elicited stronger JSEs than the latter [28]. These combined findings suggest that a partner's social characteristics can modulate the JSE, and bolster the notion that there may be some intrinsically social, domain-specific processes underlying implicit mentalization, as captured by the Simon task.

In contrast, domain-general accounts posit that the JSE can be explained by non-socially-specific processes. An example of this is the spatial response coding account, which proposes that the partner in a Joint Simon task simply serves as a spatial reference frame [29,30]. In other words, this account argues that any salient entity can, regardless of its social characteristics, act as a trigger for the spatial stimulus–response compatibility effect and hence induce the JSE [31]. For example, a study demonstrated that a Japanese waving cat (a small animatronic statue) or even a metronome placed next to a participant (in place of a human partner) during a Joint Simon task elicited JSEs [32]. However, the spatial response coding account fails to explain the modulation of JSEs from social factors. In an attempt to integrate these findings, Dolk *et al*. [32] proposed the referential coding account (similarly, the theory of event coding; for an overview, see [33]).

The referential coding account posits that the cognitive representations of actions are composed of event codes [33]. These codes denote the possible perceivable effects that would result from performing the cognitively represented action. These perceivable effects could manifest as different visual, auditory, and proprioceptive sensory inputs or even object-specific effects such as speed and orientation [34]. For example, the action of squeezing a yellow rubber duckie may include perceivable effects—and subsequently event codes—such as ‘yellow’, ‘right hand’, ‘quack’, etc. The referential coding account does not intrinsically distinguish between self- and other-generated event codes, nor between the actions represented by those respective codes. It may therefore be extrapolated that the event codes for generating an individual's own actions and a partner's actions during a shared task will overlap to a large degree because the desired goal criteria (i.e. desired action outcomes) are highly similar [35].

It is possible to apply the referential coding account to explain the Simon task and implicit *mentalizing*: a participant sitting on the left, tasked with pressing a response button when a certain colour appears, will necessitate numerous event codes, such as ‘left response location’, ‘fast action’, ‘clicking sound’, ‘index finger’, ‘human action’, etc. To determine the correct course of action, Dolk *et al*.'s [36] referential coding account argues that task-relevant codes must be selected out of all these event codes for a response. During an Individual go/no-go Simon task, there exists only one perceived self-generated action, thus creating no conflict (and no SCEs). In contrast, during a Joint Simon task, there exists an additional perceived action because the referential coding account does not distinguish between self-generated events and perceived events, with the latter events being generated by the partner in this scenario. This effectively doubles the amount of concurrently activated event codes. Given the many similar event codes shared between the self and the partner (e.g. ‘fast action’, ‘clicking sound’, etc.), one of the most salient discriminating features between the two is their difference in relative spatial locations. It is argued that this prompts an increased emphasis on the left versus right dichotomy, thereby reintroducing the stimulus–response spatial overlap, and eliciting the JSE. This account alone, however, is still unable to explain why social variables such as the partner's emotional state and interpersonal closeness modulate the JSE.

The crux of Dolk *et al*.'s [36] referential coding account, and the reason for its apparent ability to account for the effect of social factors on the JSE, lies in the notion that the degree of resemblance between self-related and partner-related codes (e.g. similarities/differences in the physical body, affect, ideological status, etc.) inadvertently increases/decreases the magnitude of the overlap between self-/other-event codes, with a corresponding knock-on effect on the difficulty/ease of discriminating between the codes. Under this rationale, Dolk *et al*. [36] argued that higher similarities in self-/other-event codes would be associated with more challenging action event discrimination (due to fewer differences between event codes), thereby increasing the JSE. Indeed, the previously discussed line of socially related findings (e.g. larger JSE in ingroup versus outgroup [28]) map decently onto this account, as it would be reasonable to suggest that partners who are socially closer to oneself would have more similarities than more distant partners. Furthermore, it is possible to account for findings of varying magnitudes of JSE between human(oid) and non-human(oid) partners. For example, studies have found that humanoid partners elicit larger JSEs than mechanoid robots [37] and wooden hand puppets [38]. With the notion that both social and non-social ‘partners’ can trigger the JSE, the referential coding account is able to consolidate several theoretical lines of thought and therefore provides strong evidence supporting the view that implicit *mentalizing*, as captured through the Joint Simon task, seems to be driven by domain-general mechanisms. However, there is a potentially pivotal avenue of investigation left yet unturned and unaccounted for by the referential coding account, concerning the *contents* of co-representation.

The lack of agreement throughout the literature may have partially arisen from the limits of one-dimensional behavioural measures (e.g. reaction times (RTs) and response accuracies) within the confines of the Simon task, which precludes probing into the contents of co-representation. Crucial clues to the domain specificity of implicit *mentalizing* may be discoverable through the examination of *what* is being co-represented (if at all). Some prior studies have investigated this question within the context of various joint tasks by testing participants' memory of their partner's stimuli (e.g. [39,40]; for a review, see [41]). For example, Eskenazi *et al*. [42] tested participants with a computerized word categorization task where they were tasked with responding to certain categories of words (e.g. only to plant-related words, but not words of household items), both individually and in pairs. In the Individual go/no-go task, each participant was responsible for one-word category (out of three). In the Joint task, each of the two participants was responsible for one category, with the third-word category as control. Afterwards, a surprise free-recall task was presented in which participants had 2 min to list as many previously seen words as possible. Performance in the words assigned to oneself (Self-assigned) was responded to with similar speed and accuracy between Individual and Joint tasks. However, even though participants were not told to pay attention to their partner's words, participants were significantly more accurate at remembering words that their partner had responded to (Partner-assigned), than those that had not been responded to (i.e. Control). These findings suggest that the contents of a partner's task may be non-consciously and spontaneously encoded during a shared task above baseline incidental memory, even when not required or instructed to do so. However, prior studies like this have involved the intentional processing of the stimuli that will be tested in the Recognition task (e.g. categorizing words). According to the referential coding account, these explicit categorization choices may require disambiguation between self-/other-event codes, thereby introducing non-socially-related attentional and memory biases towards the Partner-assigned stimuli. This design may have therefore potentially confounded the referential coding account-driven memory biases with possible partner-driven effects.

## 2. Present study

To overcome this limitation, the present study further assessed the contents of co-representation during task sharing by substituting geometric stimuli used in typical Joint Simon tasks with unique images (in our case, animal silhouettes). Critically, participants were unaware of the significance of the animal silhouettes—they were only tasked with attending to the stimuli's colour, akin to a typical Simon task. We implemented two task conditions: the Joint Simon task (two participants, each responsible for half the Simon task) and the Individual go/no-go task. A surprise Recognition task was subsequently appended to the Simon task, in which we measured the participants' incidental memory of the unique set of stimuli presented during the Simon task. We then compared the participants’ recognition accuracy of stimuli between two assignments; that is, between stimuli of colours that were relevant to themselves (i.e. of one's assigned colour; Self-assigned) versus stimuli relevant only to their partner in the Joint Simon task (i.e. not of one's assigned colour; Partner-assigned); or, in the Individual go/no-go task, relevant to nobody (Self-assigned versus Not-assigned). Finally, recent studies have demonstrated that higher interpersonal closeness with one's partner is a positive predictor of SCEs in Joint Simon tasks [26,43]. To potentially replicate, identify, and control for such factors, measures of interpersonal closeness were included.

In sum, the present study has four main aims: (1) to ascertain if the present novel Joint Simon task adaptation is able to elicit the JSE; (2) to examine the contents of co-representation during the Joint Simon task via a surprise incidental Recognition task, which will in turn address the domain specificity debate around implicit *mentalizing*; (3) to examine the effect of interpersonal closeness on JSE and image recognition performance; and (4) to exploratorily investigate if the magnitude of SCEs in the Simon task predicts task co-representation and memory encoding during task sharing.

### 2.1. Hypotheses

**H1:** Given the similarities between the present methodology and a typical Joint Simon task, we predict that the present adapted version of the Joint Simon task will elicit the JSE in the form of an interaction between Compatibility and Task Condition. Specifically, we predict that participants in the Joint Simon task will display a greater SCE than participants in the Individual go/no-go task—participants in the Joint Simon task will be slower and less accurate when reacting to incompatible trials.

**H2:** In the surprise Recognition task, we predict a similar line of results to previous research on incidental memory during task sharing [42], that is, stimuli attended to by a task partner will be more accurately remembered than control stimuli. Specifically, we predict that image recognition accuracy for Partner-assigned stimuli in the Joint Simon task will be superior to the equivalent Not-assigned stimuli in the Individual go/no-go task. In contrast, we predict that the recognition accuracy for Self-assigned stimuli will remain similar across Individual go/no-go and Joint Simon tasks.

**H3:** Considering past findings [26,43], we predict that interpersonal closeness ratings will be predictive of the degrees to which participants represent their partners' mental states in the Simon task and the Recognition task.

**H3a:** In the Simon task, we predict that participants with higher interpersonal closeness ratings of their partners will display greater magnitudes of SCEs than participants with relatively lower ratings. Specifically, the former will be slower and less accurate when reacting to incompatible trials.

**H3b:** In the surprise Recognition task, we predict that participants with higher interpersonal closeness ratings of their partners will recognize their Partner-assigned stimuli more accurately than participants with lower interpersonal closeness ratings of their partners.

**H4:** The magnitude of SCEs recorded in the Joint Simon task is representative of the extent to which a participant was influenced by the presence of their partner. It is therefore possible that the magnitude of SCEs may serve as a proxy for the extent of task co-representation and memory encoding between participants. We therefore exploratorily hypothesize that SCE magnitude will predict image recognition accuracy, even when accounting for the effects of interpersonal closeness.

### 2.2. Implications

As set out in H1, if the present novel adaptation of the Joint Simon task is capable of eliciting the JSE, it will unlock the potential for new methods of studying co-representation, and further inform the domain-specificity debate. Evidential support for H2 will add an interesting counter-perspective to the referential coding account, as an enhanced incidental memory of the partner's stimuli (exclusively in the Joint Simon task) may suggest that a partner's stimuli are co-represented and spontaneously encoded within the participant's memory. This will be above and beyond what can be explained by the low-level referential coding account—since the tested measure will be incidental memory, there will be no explicit action or choice, nor the necessity for the direct involvement of self-/other-event codes. Hence, such a finding will point towards distinctly social aspects of implicit *mentalizing* and provide support for the domain-specific social account. Contrarily, the robust absence of support for H2 (i.e. recognition accuracy of Partner-assigned stimuli is not better than Not-assigned stimuli), along with the validation of H1 (i.e. a JSE is found), would be consistent with the referential coding account. This finding will therefore bolster the notion that the emergence of the JSE is underlaid by domain-general mechanisms, suggesting that implicit *mentalizing* may not be implicated.

In terms of H3, if higher ratings of interpersonal closeness are found to be associated with heightened JSEs and partner-driven memory effects, it will support the domain-specific notion that socially related factors are intertwined with implicit *mentalizing* during task sharing. Lastly, it is also possible that results between the hypotheses H2/H3 are not consistent. For example, there could be support only for H3, but not H2 (or vice versa); that is, where Task Condition has no significant effect on recognition accuracy between Assignment levels, yet interpersonal closeness predicts recognition accuracy of Partner-assigned stimuli. This line of results may suggest that there exists an interpersonal closeness-modulated partner-driven effect on incidental memory (and therefore possibly implicit *mentalizing*); however, this effect may be too small to be detectable when comparing the Individual and Joint task conditions. Such a result will still provide support for the social, domain-specific account, albeit at a weaker level than if H2 and H3 were consistent. Alternatively, if there is only support for H2, but not H3, this may suggest that the recognition advantage for Partner-assigned stimuli in the Joint Task Condition is primarily driven by non-socially-related factors, thereby supporting the domain-general account.

Finally, in relation to the exploratory H4, if results indicate that SCE magnitudes in the Simon task significantly predict image recognition accuracy of Partner-assigned stimuli when accounting for interpersonal closeness, this may support the notion that participants who have been more influenced by the presence of their partner also have memory records that reflect their representation of the partner-relevant items in a joint task. Such a result will provide evidence for task co-representation during shared tasks, and substantiate the domain-specific, social account of the JSE and implicit *mentalizing*. Alternatively, a robust absence of support for H4 may imply that the effect from the presence of a partner does not influence the incidental memory of partner-relevant items. These results would suggest that participants do not necessarily represent their partners' visual stimuli during task sharing, and therefore point towards a domain-general account of the JSE. For a tabularized summary, see table 1.

**Table 1.** Summary of research questions, analysis plan and outcome interpretations.

research question	hypotheses	analysis plan	interpretation given different outcomes
1. does the present adapted version of the Simon task elicit JSE?	given the similarities between the present methodology and a typical Simon task, we predict that the present adapted version of the Simon task will elicit JSE, and will produce the signature effect in behavioural response in the form of an interaction between Task Condition and Compatibility	linear mixed-effects model with trial-level RT in the Simon task as the dependent variable, and fixed effects of Compatibility (Compatible versus Incompatible) and Task Condition (Joint Simon versus Individual go/no-go). Maximal theoretical random effects for Item and Participant will be included (for specifics, see Planned Analysis section)	a significant interaction effect between Task Condition and Compatibility, with the Compatibility effect (i.e. SCE) being stronger in the Joint Simon task than the Individual go/no-go task would suggest that these novel variations of the Individual go/no-go versus Joint Simon task replicate previous behavioural findings and elicit JSE
			if the interaction between Task Condition and Compatibility is not found, we will consult the *BF*_10_ associated with this effect to determine the robustness of the null effect. If the null interaction effect is robust then interpretation will be made on the basis on any significant main effects. Minimally, a main effect of Compatibility is expected to evidence that participants have engaged with the task as we intended
2. does implicit *mentalizing* during joint action involve the co-representation of a partner's perspective content?	we predict that image recognition accuracy of the Self-assigned stimuli will remain similar across the Individual go/no-go task and the Joint Simon task, whilst Partner-assigned stimuli will be better remembered in the Joint task than the equivalent not-assigned stimuli in the Individual go/no-go task	generalized linear mixed-effects model with binary accuracy in the Recognition task as the dependent variable, and fixed effects of Task Condition (Joint Simon versus Individual go/no-go) and Assignment (Self-assigned versus Other-assigned). Maximal theoretical random effects for Item and Participant will be included	if the hypothesized above-normal incidental memory of the partner's stimuli (manifesting as an interaction between Task Condition and Assignment) is found, it may suggest that a partner's stimuli are somehow co-represented and spontaneously encoded within the participant's memory. Such a finding will suggest that there may be distinctly social aspects of implicit *mentalizing* and provide support for the domain-specific social account
			contrarily, a robust absence of this finding would be consistent with the referential coding account, and therefore further bolster the notion that the emergence of the JSE is underlaid by domain-general mechanisms
3. does interpersonal closeness positively predict the magnitude of the JSE and the amount of perspective content co-representation during task sharing?	we predict that participants with higher interpersonal closeness ratings of their partners will display larger SCEs in the Simon task and heightened image recognition accuracy of their partner's stimuli in the Recognition task when compared against those with lower interpersonal closeness ratings of their partners	linear mixed-effects model with trial-level RT in the Joint Simon task as the dependent variable, and fixed effects of Compatibility (Compatible versus Incompatible) and the IOS scale	if interpersonal closeness is positively associated with the magnitude of the JSE and visual content co-representation (manifesting as an interaction between Compatibility and IOS in the Simon task, and higher image recognition accuracy of Partner-assigned stimuli in the Recognition task respectively) during task sharing, it will support the notion that socially related factors are intertwined with implicit *mentalizing* during task sharing, thus substantiating the domain-specific account
		a generalized linear mixed-effects with trial-level recognition accuracy in the Recognition task from participants who took part in the Joint Simon task as the dependent variable, and fixed effects of Assignment (Self-assigned versus Partner-assigned) and the IOS scale. Maximal theoretical random effects for Item and Participant will be included for both maximal models	alternatively, a robust absence of such an effect may suggest that socially specific processes for implicit *mentalizing* during task sharing do not underly the JSE, and hence support the domain-general account
4. exploratory: does the magnitude of SCEs in the Simon task predict task co-representation and memory encoding during task sharing?	we predict that participants with higher SCE magnitudes in the Simon task will perform better in image recognition accuracy of partner-relevant stimuli	a generalized linear mixed-effects with trial-level recognition accuracy in the Recognition task from participants who took part in the Joint Simon task as the dependent variable, and fixed effects of Assignment (Self-assigned versus Partner-assigned) and SCE magnitude. Maximal theoretical random effects for Item, Participant, and the IOS scale will be included	if SCE magnitudes predicts image recognition accuracy of partner-assigned stimuli, this may suggest that the degree of influence from the presence of their partner may also have affected incidental memory of partner-relevant items in a joint task. Such a result will provide evidence for task co-representation during shared tasks, and substantiate the domain-specific, social account of the JSE and implicit *mentalizing*
			alternatively, a robust absence of a relationship between SCE magnitude and image recognition accuracy of partner-assigned stimuli may imply that the effect from the presence of a partner does not influence the incidental memory of partner-relevant items. These results would suggest that participants do not necessarily represent their partners’ visual stimuli during task sharing, and therefore point towards a domain-general account of the JSE

## 3. Method

The approved Stage 1 protocol can be found on OSF Registries (https://osf.io/m8tgy).

### 3.1. Participants

Sixty-two participants provided informed consent to participate in the experiment; the final sample used for analysis, apart from exclusions, was 52 participants (*M*_age_ = 18.8 years, *s.d.*_age_ = 2.26; 43 females). Participants were over 16 years of age, recruited via a research participation SONA system or through opportunistic recruitment around the university campus. All participants had normal or corrected-to-normal vision and normal colour vision. Participants were compensated with course credits or paid £5 for their time. Ethical approval was granted by the Lancaster University Faculty of Science and Technology Research Ethics Committee (reference: FST-2022-2186-RECR-2). The 10 participants who were excluded as stipulated in the Data processing and exclusion section were replaced with new participants.

### 3.2. Sample size determination

The present study aimed to capture the critical JSE as a mixed interaction between Compatibility (Compatible versus Incompatible) and Task Condition (Joint Simon versus Individual go/no-go). Past studies of the JSE have obtained medium-to-large interaction effect sizes (e.g. [26,44]). An *a priori* power analysis was performed using the Power ANalysis for GEneral Anova (PANGEA, v. 0.2) [45] to estimate the participant sample size required to detect a similar interaction. Due to the novel adaptations made to the Simon task (thus possibly attenuating the strength of previously found effects) and the additional Recognition task, a conservative-leaning effect size estimate was used to calculate the sample size required for the current study. We inputted Compatibility and Task Condition as fixed effects, and Stimuli (i.e. unique animal silhouettes) and Participants as random effects (full details available on OSF: https://osf.io/zgdnv). With power set to 0.9 and effect size *d* set to 0.4, the projected sample size needed to detect a medium-small effect size, 2 × 2 within–between interaction was approximately *N* = 72 (i.e. *n* = 36 per Task Condition).

Following best-practice guidelines for implementing Bayesian stopping rule [46], we used a sequential design with maximal *n*, such that we collected data until either of the following two conditions were met: (1) a compelling Bayes factor of 3+ supporting either the null or alternative model for the key JSE is reached (for more information about Bayesian statistics, see the Analytic approach section below); or (2) the maximum feasible sample size (due to constraints in resources associated with recruitment) is reached. To inform the upper/lower limits of monitoring our Bayesian stopping rule, we calculated the projected *N* required at incremental points above/below effect size *d* = 0.4 (lower bound: *d* = 0.45; upper bound: *d* = 0.35). In other words, we began monitoring the Bayesian stopping rule when our sample size was capable of detecting an interaction effect with a size of *d* = 0.45. Power analysis indicated that this will require *n* = 25(+1 to make it even for the Joint Task Condition) per Task Condition, for a total of *N* = 52. We set our maximum feasible sample size at the point where we can detect an effect size of *d* = 0.35, which will require *n* = 59(+1 to make it even), for a total of *N* = 120.

### 3.3. Stimuli and materials

The online survey software Qualtrics [47] was used to provide participants with information and consent forms, plus obtain demographic information and (for participants in the Joint Simon task) interpersonal relationship scores (see https://osf.io/mus39 for all experimental materials, questionnaires, data and analysis scripts). The Simon and Recognition Tasks were run using PsychoPy [48] on three iMac desktop computers with screen sizes of 60 cm by 34 cm and screen resolutions of 5120 × 2880 at 60 Hz. Responses to the Simon task were recorded using individual numeric keypads.

**Figure 1.**
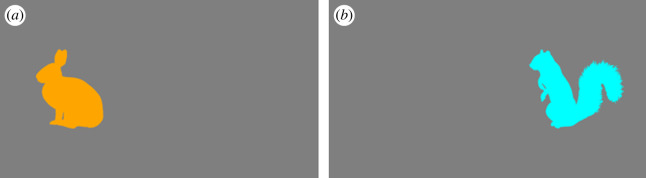
Example stimuli in the adapted Simon task. Note that (*a*) contains a screenshot of the adapted Simon task in which the orange stimulus appears on left, whilst (*b*) depicts a blue stimulus appearing on the right.

Sixty-four animal silhouettes were chosen from PhyloPic [49], an online database of taxonomic organism images, freely reusable under a Creative Commons Attribution 3.0 Unported licence. All images were resized and standardized to fit within an 854 × 854-pixel square. The animal silhouettes were recoloured to be entirely in either blue (hexadecimal colour code: #00FFFF) or orange (#FFA500). In each trial, the animal silhouettes were displayed either 1440 pixels on the left or the right from the centre of the screen (for example, see figure 1).

### 3.4. Design

#### 3.4.1. Simon task

**Figure 2.**
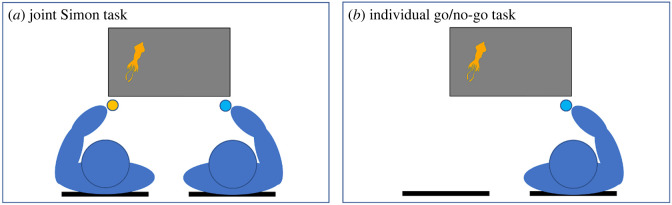
Experimental setup of the Simon task.

For the Simon task, a 2 × 2 mixed design was employed, with Compatibility (Compatible versus Incompatible) as a within-participant variable and Task Condition (Joint Simon versus Individual go/no-go) as a between-participant variable. Participants were first individually directed to computers running Qualtrics to read and sign information and consent forms, and to provide demographic information. Afterwards, participants were guided to sit at a third computer. Participants sat approximately 60 cm (diagonally, approx. 45° from the centre of the screen) away from the computer screen either on the left or right side, each with a number keypad in front of them (figure 2). They were instructed to use their dominant hand on the button. In the Joint Simon task, each pair of participants sat side-by-side, approximately 75 cm beside their partner. In the Individual go/no-go task, an empty chair was placed in an equivalent location next to the participant.

Each participant was assigned a colour (either blue or orange) to respond to. Participants were instructed to ‘catch’ the animal stimuli by pressing a button when an animal silhouette of their assigned colour appeared on the computer screen.^1^ Participants were told to push the button as quickly and accurately as possible. Participants were not otherwise instructed to pay specific attention to any of the animal species, nor the location (left versus right) that any stimulus appears; the focus was solely on the animal silhouettes' colour. Crucially, participants were unaware of the Recognition task which followed. Thirty-two out of the set of 64 animal silhouettes were displayed to participants during the Simon task. The 32 animal silhouettes were further divided in half and matched to each of the two colours, such that each participant was unknowingly assigned 16 animal silhouettes in their respective colours. The remaining 32 animal silhouettes were used as foils in the Recognition task. Participants' sitting location (left versus right), stimuli colour (blue versus orange), stimuli presentation position (left versus right), and animal silhouettes to be presented (as stimuli in the Simon task versus as foils in the Recognition task) were counterbalanced between participants.

#### 3.4.2. Surprise recognition task

For the Recognition task, a 2 × 2 mixed design was employed, with Assignment (Self-assigned versus Partner-assigned in the Joint Simon task; Self-assigned versus Not-assigned in the Individual go/no-go task) as a within-participant variable and Task Condition (Joint Simon versus Individual go/no-go) as a between-participant variable. Assignment refers to whether an animal silhouette was presented in a colour that required a response from the participants (i.e. Self-assigned) or their partner (i.e. Partner-assigned) during the Simon task. In an Individual go/no-go task, the equivalent latter level referred to the colour that did not require a response from the participant (i.e. Not-assigned). Hereafter, when referring to the Assignment condition in the context of analyses, we will group the two equivalent levels into ‘Self-assigned’ and ‘Other-assigned’.

### 3.5. Procedure

Participants first completed a practice section of the Simon task with circles as stimuli. In the practice section, if a participant correctly pressed their button when the stimulus of their assigned colour appeared, they were met with the text-based feedback ‘well done’. Incorrect responses (i.e. when a participant pressed their button when a stimulus not of their assigned colour appeared) or timeouts (i.e. failure to respond within 1000 ms) were met with the feedback ‘incorrect, sorry’ or ‘timeout exceeded’ respectively. There was no feedback in the main experiment. When each participant completed a minimum of five trials and achieved 90% running accuracy, they were allowed to proceed to the main experiment. The main experiment consisted of four experimental blocks, each containing 32 trials (corresponding to the 32 chosen animal silhouettes), totalling 128 trials for the full experiment. A mandatory 10 s break was included at the half-way point of the experiment. Half of the trials in each block were compatible trials (the coloured stimulus and its correct corresponding response pushbutton were spatially compatible), while the remaining half were incompatible trials. Each block contained 8 compatible trials and 8 incompatible trials for each colour.

**Figure 3.**
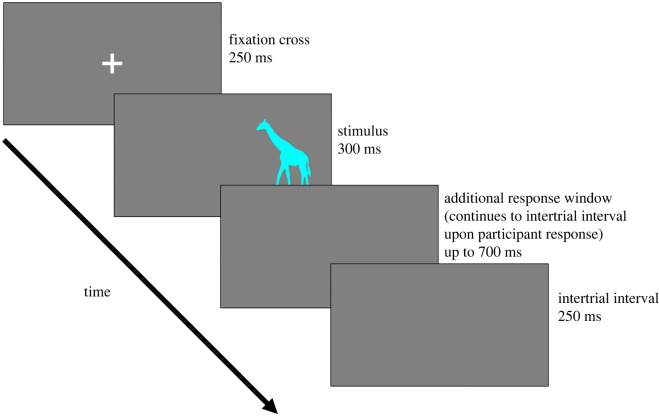
Trial sequence diagram of the Simon task. The stimulus always appeared for exactly 300 ms; trials only continued to the intertrial interval after at least 300 ms had elapsed. For trials where participants responded before the stimuli disappears (i.e. within 300 ms of stimuli onset), the ‘additional response window’ was skipped; such trials proceeded immediately to the intertrial interval.

As shown in figure 3, each trial began with a fixation cross in the centre of the screen presented for 250 ms. Following this, a colour stimulus (a circle in the practice trials, an animal silhouette in the main experiment) appeared on either the left- or right-hand side of the screen for exactly 300 ms, disappearing afterwards into a blank screen. Participants had another 700 ms to respond (i.e. 1000 ms in total post-stimuli onset) before a trial was timed out and continued to the intertrial interval. After a participant responds, provided that at least 300 ms post-stimuli onset has elapsed, the trial continued to the intertrial interval. Following designs of similar studies (e.g. [50]), no-go trials in the Individual go/no-go Task Condition continued to the intertrial interval 300 ms post-stimuli onset. A 250 ms intertrial interval (blank screen) was implemented to help participants visually delineate between trials. Response accuracy and reaction time (time elapsed between stimulus onset and a button press) on each trial were recorded as response variables.

Regardless of participants' response time, each stimulus remained on the screen for the full 300 ms. This was to ensure that each animal silhouette was displayed for an equal length of time. This was important so as not to bias the incidental memory of participants towards trials wherein one participant was slower to respond, which would have therefore kept a stimulus on screen for longer and disproportionally encouraged encoding.

After completing the Simon task, participants were each guided back to their individual computers which they had initially used to give consent and demographic information, so as to minimize bias from spatial context on memory. Using a PsychoPy programme, participants were shown 64 black-and-white animal silhouettes one-by-one and were asked two questions: (1) ‘Do you recall seeing this animal in the task before?’, with binary ‘yes’ or ‘no’ response options; and (2) ‘How confident are you in your answer above?’, with a 7-point Likert scale between 1 = *extremely unconfident* to 7 = *extremely confident* as response options. For both questions, participants used a computer mouse to select a response option on the screen. Participants were instructed that it does not matter what colour the animal silhouettes appeared in during the preceding (Simon) task—so long as they remember having seen the silhouette at all, they were asked to select ‘yes’. There was no time limit on this task. Of the 64 animal silhouettes that were presented, 32 were seen in the Simon task, while an additional 32 animal silhouettes served as foils in this Recognition task. The presentation order of the animal silhouettes was randomized for every participant. Participants' responses to the two aforementioned questions were recorded as key response variables.

At the end of the study, participants were asked several post-test questions which, depending on their answers, would lead to further questions. For example, they were asked whether they had any suspicions of what the study was testing, or whether they paid specific attention to, and/or memorized the animal species shown in the Simon task on purpose. The latter questions served to identify whether participants intentionally memorized the animal silhouettes, which would undermine the interpretability of the data collected in the Recognition task.

**Figure 4.**
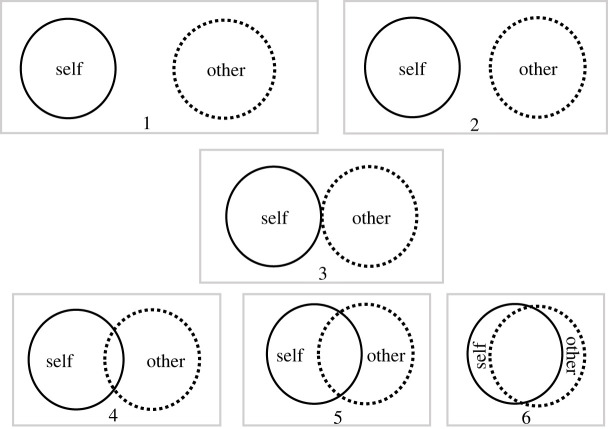
Inclusion of Other in the Self (IOS) scale.

In following the steps of Shafaei *et al*. [26], participants in the Joint Simon task were also asked to individually rate their feelings of interpersonal closeness to their task partner via the Inclusion of the Other in the Self (IOS) scale [51], which consisted of pictographic representations of the degree of interpersonal relationships. Specifically, as shown in figure 4, the scale contains six diagrams, each of which consisted of two Venn diagram-esque labelled circles which represent the ‘self’ (i.e. the participant) and the ‘other’ (i.e. the participant's task partner) respectively. The six diagrams depict the circles at varying levels of overlap, as a proxy measure of increasing interconnectedness. Participants were asked to rate which diagram best described the relationship with their partner during the study. This served as a measure of interpersonal closeness.

## 4. Analysis strategy

### 4.1. Analytic approach

To retain the most amount of information (i.e. preserving trial-level data), and simultaneously account for possible random effects from repeated by-participant and by-item measurements, (generalized) linear mixed-effects models were employed. The computer language system focused on statistical analysis and data visualization R (v. 4.2.1 [52]) was accessed via the integrated development environment R Studio (v. 2022.7.1.554 [53]).

A maximal-to-minimal design-driven model structuring technique was adopted (in contrast to data-driven effect structuring; for more information, see [54]). This technique entails the assembly of a maximal random effects model justified by experimental design. In practice, this involved first specifying all theoretically relevant main effects and interactions as fixed effects. For continuous variables (e.g. reaction time), a linear mixed effects model was fitted using the lmer() function from the R package lme4 [55]. For categorical variables (e.g. binary accuracy), a generalized linear mixed effects model was fitted using the glmer() function instead. Then, for every sampling unit (i.e. item or participant) with repeated measures, a random intercept was added. Additionally, random slopes (and all associated interaction effects) for all within-participant factors were added, altogether of which form the maximal justifiable model.

In the case of model non-convergence, the simplification of the model's random effects structure was required. This was systematically achieved through constraining all covariance parameters to zero, then eliminating the random effects which explained (near-)zero variance and/or had (near-)perfect intercorrelations. This process was repeated until the model reached convergence, thereby fitting a maximal converging model. Nested model comparisons were then conducted using likelihood ratio tests (LRTs) through the anova() function to determine which fixed effects and interactions were statistically significant. This was achieved by removing a single main effect or interaction fixed effect from the maximal converging model and comparing that nested model's fit against the full model. For the full protocol for model structuring, see [56]. Details of all modelling decisions were also annotated in the R script used for analysis. As advised by best practice guidelines for linear mixed model reporting [57], a full table summary is provided for every major fitted model.

Bayes information criteria (BIC) [58] were obtained during LRT model comparisons and were further computed into Bayesian factors [59]. Bayes factors are especially relevant when considering null effects (as determined by frequentist measures, i.e. *p* > 0.05), because they can provide probabilistic evidence for the alternative model relative to the null model, as denoted by *BF*_10_. Specifically, according to guidelines by Wetzels *et al*. [60], *BF*_10_ < 1/3 represents evidence in support of the null model over the alternative model, and *BF*_10_ > 3 represents evidence for the alternative model over the null model. For this reason, *BF*_10_ parameters were reported alongside all fixed effects in the model summaries.

### 4.2. Data processing and exclusion

To prepare the dataset for analysis using R, individual data outputted from PsychoPy and Qualtrics were combined into master data frames. Participants who stated that they intentionally memorized the animal silhouettes during the Simon task by using specific memory strategies were excluded, resulting in the exclusion of one participant. We would have excluded participants who failed to respond correctly to at least 90% of trials (i.e. 115 out of 128 trials) in the Simon task, in line with the highly accurate performances observed in previous studies (e.g. [61–63]). This criterion would have identified and excluded participants who did not undertake the task as intended; however, in the present sample, zero participants had to be excluded under this criterion. For the response time data obtained from the Simon task, from a total of 3343 trials, 28 (0.84%) were excluded due to incorrect responding and 6 (0.18%) were excluded due to timing out. To match previous studies (e.g. [43]), we did not analyse trials with outlying RTs which were three or more s.d. away from each participant's overall RT mean, resulting in the further exclusion of 40 (1.21%) of 3309 trials. We excluded participants from Recognition accuracy analyses if they responded incorrectly at a level that was significantly below chance, resulting in the exclusion of five participants. A Binomial probability calculation indicates that participants must respond correctly to at least 39 out of 64 total trials to be significantly above chance.

Deviation contrast coding was applied to all categorical predictor variables, such that every fixed effect level was compared against a grand mean. Following prior studies (e.g. [26,43,64]), the IOS scale of interpersonal closeness was treated as a continuous variable.

### 4.3. Planned analyses

#### 4.3.1. Simon task

To test the hypothesis H1 and identify whether the JSE (i.e. the interaction between Task Condition and Compatibility, where stronger effects of Compatibility are found in the Joint Simon over Individual go/no-go task) can be elicited in the present adapted Simon task variant, a linear mixed-effects model was computed. The model contained fixed effects of Compatibility (Compatible versus Incompatible; coded as 0.5 versus −0.5) and Task Condition (Joint Simon versus Individual go/no-go; coded as −0.5 versus 0.5), both as main effects and interactions in all models. The initial maximal model included Participant (i.e. different participants) and Item (i.e. the 32 different animal silhouette images) as random intercepts. Additionally, by-Participant random slopes for Compatibility and by-Item random slopes for Task Condition, Compatibility, and their interaction were added.

To test the hypothesis H3a and identify whether interpersonal closeness modulates the magnitude of SCEs, and if interpersonal closeness itself accounts for significant variance in response time, another linear mixed-effects model was computed with Joint-only participants (because only participants in the Joint task were asked to give closeness ratings). The model contained fixed effects of Compatibility (Compatible versus Incompatible) and the IOS scale, both as main effects and interactions in all models. The initial maximal model included Participant and Item as random intercepts. By-Participant random slopes for Compatibility and by-Item random slopes for IOS, Compatibility, and their interaction were added.

#### 4.3.2. Recognition task

Addressing the hypothesis H2 that recognition performance may be heightened for animal silhouettes assigned to the participant's partner in the Joint Simon task (but not the equivalent ‘Not-assigned’ stimuli in the Individual go/no-go task), a generalized linear mixed-effects model was fitted to predict binary recognition accuracy. Because the primary area of interest was in performance differences between animal silhouettes assigned to either the self or other during the Simon task, only the animal silhouettes that were seen during the Simon task were analysed (i.e. foils were excluded). Task Condition (Joint Simon versus Individual go/no-go; coded as −0.5 versus 0.5), Assignment (Self-assigned versus Other-assigned; coded as 0.5 versus −0.5), and their interactions were added as fixed effects. The initial maximal model included Participant and Item as random intercepts, in addition to by-Participant random slopes for Assignment and by-Item random slopes for Task Condition.

To verify that response accuracy covaried with response confidence equally across all factors (i.e. to check that there are no specific factor levels where participants were confident in their responses, whilst performing particularly poorly, or vice versa), another generalized linear mixed-effects model was fitted to predict binary recognition accuracy. Response Confidence (1–7 points on a Likert scale), Task Condition (Joint Simon versus Individual go/no-go), and Assignment (Self-assigned versus Other-assigned) were included as fixed main effects, in addition to a three-way interaction between the three main effects, plus all lower-order interactions. The maximal model contained Participant and Item as random intercepts and Assignment as a by-Participant random slope.

#### 4.3.3. Interpersonal closeness

To test the hypothesis H3b, a generalized linear mixed-effects model was fitted to ascertain whether interpersonal closeness significantly contributes to variance in predicting recognition accuracy in the Joint Simon task. The model contained fixed effects of Assignment (Self-assigned versus Other-assigned) and the IOS scale, as both main effects and interactions in all models. The initial maximal model included Participant and Item as random intercepts. By-Participant random slopes for Assignment and by-Item random slopes for IOS were added.

#### 4.3.4. Exploratory effect of SCE magnitude

To test the exploratory hypothesis H4 that the magnitude of SCEs may predict image recognition performance, a generalized linear mixed-effects model was fitted to predict binary recognition accuracy. Scaled participant-level SCE magnitudes were calculated by subtracting each participant's mean RT for Compatible trials from their mean RT for Incompatible trials in the Simon task, then dividing by the sum of the Compatible and Incompatible trials. This scaling method accounted for each participant's baseline reaction speed [65]. The model contained fixed effects of Assignment (Self-assigned versus Other-assigned) and SCE magnitudes, as both main effects and interactions in all models. The initial maximal model included Participant, Item, and the IOS scale as random intercepts. By-Participant random slopes for Assignment, and by-Item random slopes for IOS, were added.

## 5. Results

Unless stated otherwise, all reported null results were substantiated by both frequentist (*p* > 0.05) and Bayesian statistics (*BF*_10_ < 1/3), providing converging evidence in support of the null model in the respective analyses. In all mixed-effects model summaries, *p*-values for fixed effects were calculated using Satterthwaites approximations. Confidence intervals were calculated using the Wald method. Marginal pseudo-*R*^2^ represents the proportion of variance explained by fixed effects, while conditional pseudo-*R*^2^ represents the variance explained by the entire model (i.e. both fixed and random effects).

### 5.1. Simon task

#### 5.1.1. Hypothesis H1

**Figure 5.**
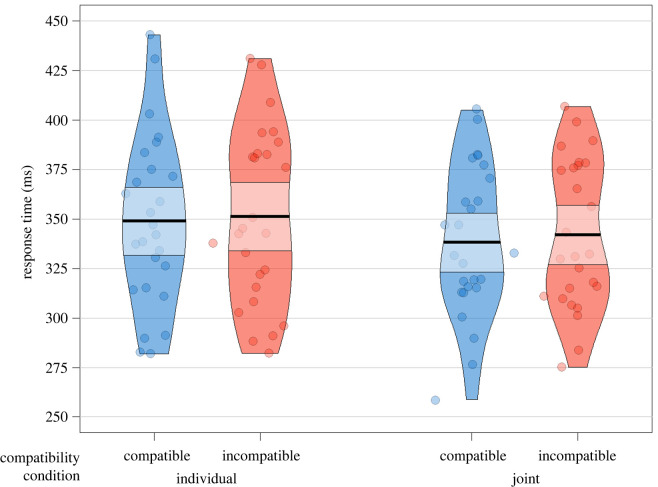
Simon task RT in milliseconds across Task Condition and Compatibility. Every dot corresponds to each participant's mean RT. Bold horizontal lines refer to mean RT on that factor level; light-coloured bands represent the 95% confidence intervals.

The results of the linear mixed-effects model predicting the effects of task condition and trial compatibility on RT in the Simon task are summarized in table 2. No significant main effects of Task Condition or Compatibility were found. Furthermore, the key interaction effect was not significant, providing evidence against H1. Table 3 provides descriptive statistics, and figure 5 depicts the RT results of the Simon task regarding hypothesis H1.

**Table 2.** Summary of the final mixed-effects model of Simon task RT, task condition and compatibility effects. Model equation: RT∼Condition * Compatibility + (1 + Compatibility | Subject) + (1 | Item).

fixed effects						
	Est/*β*	s.e.	95% CI	*t*	*p*	*BF*_10_
intercept	339.35	5.53	[328.52, 350.18]	61.42		
condition	1.29	11.05	[−20.37, 22.95]	0.12	0.907	0.018
compatibility	−2.33	1.78	[−5.82, 1.15]	−1.31	0.193	0.041
condition × compatibility	−3.92	3.56	[−10.89, 3.05]	−1.1	0.273	0.032
	random effects					
	variance	s.d.				
subject (intercept)	1554.79	39.43				
compatibility | subject	33.62	5.80				
	model fit					
pseudo-*R*^2^	marginal	conditional				
	0.01	0.43

**Table 3.** Means and standard deviations of Simon task RT in milliseconds across task condition and compatibility.

measure	compatible		incompatible	
	*M*	s.d.	*M*	s.d.
Individual go/no-go	338.51	40.16	338.89	43.09
Joint Simon	337.90	40.83	342.18	40.49

#### 5.1.2. Hypothesis H3a

The results of the linear mixed-effects model predicting the effects of interpersonal closeness and trial compatibility on RT in the Simon task are summarized in table 4. No significant main effects of IOS nor of Compatibility were found. Furthermore, the key interaction effect was not significant, providing evidence against H3a.

**Table 4.** Summary of linear mixed-effects model of Simon task RT, IOS and compatibility effects. Model equation: accuracy ∼IOS * Compatibility + (1 + Compatibility | Subject).

fixed effects						
	Est/*β*	s.e.	95% CI	*t*	*p*	*BF*_10_
intercept	312.11	16.52	[279.72, 344.49]	18.89		
IOS	10.66	5.68	[−0.47, 21.79]	1.88	0.069	0.130
compatibility	4.02	5.68	[−7.11, 15.15]	0.71	0.481	0.032
IOS × compatibility	−3.17	1.95	[−6.98, 0.65]	−1.63	0.112	0.088
	random effects					
	variance	s.d.				
subject (intercept)	1324.93	36.40				
compatibility | subject	20.59	4.54				
	model fit					
pseudo-*R*^2^	marginal	conditional				
	0.05	0.41				

### 5.2. Recognition task

#### 5.2.1. Hypothesis H2

**Figure 6.**
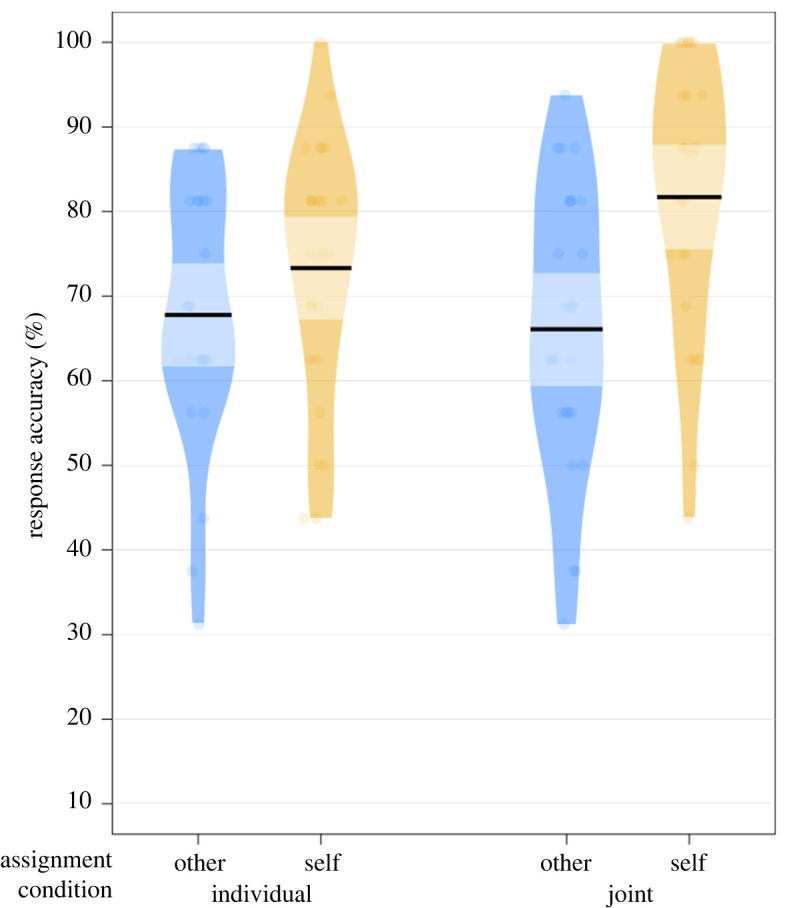
Response accuracy in Recognition task with Condition and Assignment as factors. Every dot corresponds to each participant's mean accuracy. Bold horizontal lines refer to mean accuracy on that factor level; light-coloured bands represent the 95% confidence intervals.

The results of the generalized linear mixed-effects model predicting the effects of Task Condition and stimuli Assignment on binary recognition accuracy are summarized in table 5. No significant main effect of Task Condition was found. There was a significant main effect of Assignment, where participants recognized Self-assigned stimuli more accurately than Other-assigned stimuli. Additionally, frequentist statistics indicated a significant interaction effect. This interaction was deconstructed by examining the effect of Assignment in each Task Condition individually. Specifically, Self-assigned items were recognized significantly more accurately than Other-assigned items in the Joint condition (*χ*^2^ = 17.56, *p* < 0.001, *BF*_10_ = 225.748), but not in the Individual condition (*χ*^2^ = 3.17, *p* = 0.075, *BF*_10_ = 0.169). However, the robustness of this interaction effect is constrained by the accompanying Bayesian statistic (*BF*_10_ = 0.203; *BF*_01_ = 4.926), which provides support for the null model and against H2 (i.e. suggesting that the model fits better without the interaction effect). Table 6 provides descriptive statistics, while figure 6 depicts the participants' accuracy in the Recognition task. The pre-registered quality check regarding abnormalities in response confidence ratings was computed and passed since it revealed no significant interaction effects between Response Confidence, Task Condition, or Assignment.

**Table 5.** Summary of generalized linear mixed-effects model of recognition task accuracy, condition and assignment effects. Model equation: accuracy∼Condition * Assignment + (1 + Assignment | Subject) + (1 | Item).

fixed effects						
	Est/*β*	s.e.	95% CI	*z*	*p*	*BF*_10_
intercept	1.17	0.14	[0.90, 1.45]	8.29		
condition	0.26	0.21	[−0.15, 0.67]	1.25	.213	0.053
assignment	0.74	0.16	[0.44, 1.05]	4.79	<0.001	617.26
condition × assignment	0.63	0.30	[0.04, 1.22]	2.09	0.039	0.203
	random effects					
	variance	s.d.				
subject (intercept)	0.37	0.60				
assignment | subject	0.38	0.62				
items (intercept)	0.55	0.74				
	model fit					
Pseudo-*R*^2^	marginal	conditional				
	0.04	0.26

**Table 6.** Means and standard deviations of recognition task accuracy in percentages across task condition and assignment.

measure	self-assigned		other-assigned	
	*M*	s.d.	*M*	s.d.
Individual go/no-go	73.32%	15.16%	67.79%	15.28%
Joint Simon	81.70%	15.69%	66.11%	16.79%

#### 5.2.2. Hypothesis H3b

The results of the generalized linear mixed-effects model predicting the effects of interpersonal closeness and stimuli Assignment on binary recognition accuracy in the Recognition task are summarized in table 7. No significant main effects of IOS nor of Compatibility were found. Furthermore, the key interaction effect was not significant.

**Table 7.** Summary of generalized linear mixed-effects model of recognition task accuracy, IOS and assignment effects. Model equation: accuracy∼IOS * Assignment + (1 | Subject) + (1 | Item).

fixed effects						
	Est/*β*	s.e.	95% CI	*z*	*p*	*BF*_10_
intercept	1.28	0.39	[0.52, 2.04]	3.31		
IOS	0.01	0.13	[−0.24, 0.26]	0.08	0.936	0.035
assignment	0.66	0.42	[−0.16, 1.48]	1.58	0.119	0.117
IOS × assignment	0.13	0.14	[−0.16, 0.41]	0.88	0.385	0.051
	random effects					
	variance	s.d.				
subject (intercept)	0.46	0.68				
items (intercept)	0.72	0.85				
	model fit					
pseudo-*R*^2^	marginal	conditional				
	0.05	0.30				

#### 5.2.3. Hypothesis H4

The results of the generalized linear mixed-effects model predicting the effects of SCE magnitude and stimuli Assignment on binary recognition accuracy in the Recognition task are summarized in table 8. No significant main effect of SCE was found. Furthermore, the key interaction effect was not significant. However, akin to the results in the analysis for H2, there was a significant main effect of Assignment, where participants recognized Self-assigned stimuli more accurately than Other-assigned stimuli.

**Table 8.** Summary of generalized linear mixed-effects model of recognition task accuracy, SCE and assignment effects. Model equation: accuracy∼SCE * Assignment + (1 + Assignment | Subject) + (1 | Item).

fixed effects						
	Est/*β*	s.e.	95% CI	*z*	*p*	*BF*_10_
intercept	1.39	0.22	[0.97, 1.82]	6.39		
SCE	0.00	0.01	[−0.03, 0.02]	−0.29	0.777	0.036
assignment	1.10	0.26	[0.59, 1.61]	4.21	<0.001	77.265
SCE × assignment	0.01	0.02	[−0.02, 0.05]	0.70	0.495	0.044
	random effects					
	variance	s.d.				
subject (intercept)	0.54	0.74				
assignment | subject	0.53	0.73				
items (intercept)	0.76	0.87				
	model fit					
pseudo-*R*^2^	marginal	conditional				
	0.05	0.30				

### 5.3. Unplanned exploratory analysis

Due to the null result in H1, we subjected our data to the analytical strategy typically employed in previous studies demonstrating the JSE (e.g. [26,43]). This serves to identify whether the present null findings were due to differences in the analytical approaches employed (i.e. trial-level mixed-effects analysis versus participant-level ANOVA), or a result of other methodological differences. A two-way mixed ANOVA was computed using participant-level RT in the Simon task as the dependent variable, with Task Condition as the between-participant factor, and Compatibility as the within-participant factor. The main effect of Task Condition was not significant, *F*_1,50_ = 0.79, *p* = 0.337, *η*² < 0.001; the main effect of Compatibility was also not significant, *F*_1,50_ = 2.47, *p* = 0.122, *η*² = 0.02. Finally, the interaction effect between Condition and Compatibility was not significant, *F*_1,50_ = 0.14, *p* = 0.706, *η*² < 0.001.

## 6. Discussion

The present study investigated the domain specificity of implicit *mentalizing* by examining whether there exists evidence for co-representation during task sharing. We examined whether a novel adaptation of the Joint Simon task can be used to operationalize the degree to which participants co-represent (and therefore encode) stimuli assigned to a task partner, versus when participants have no partner (hypothesis H1). The observed results demonstrated a robust null interaction effect between Task Condition and Compatibility, suggesting that the present experimental paradigm did not elicit the JSE. The absence of a JSE precludes us from speaking definitively about the results and implications of the subsequent Recognition task. However, it is insightful to explore the broader significance of these results, specifically regarding the required conditions for the JSE to emerge, such as methodological restrictions and the effect of individual differences (or a lack thereof) on the JSE.

Under the model examining H2, we asked whether Task Condition and stimuli Assignment (Self-assigned versus Other-assigned) would predict recognition accuracy. A self-reference effect was found, where participants generally remembered Self-assigned items more accurately than Other-assigned items. This is consistent with the existing literature on the self-reference effect, which has found that participants tend to more reliably encode and recall information that is relevant to oneself, in comparison to others [66,67]. The self-reference effect is thought to be driven by the integration of multiple self-relevant stimuli into a single representation, which results in an increased ease of response and enhanced encoding of the self-relevant stimuli (in comparison to non-integrated, other-relevant stimuli) [68]. The presence of the self-reference effect in the absence of the JSE may suggest that the two effects originate from the recruitment of distinct cognitive pathways. Alternatively, the self-reference effect may simply have a larger effect size than the JSE and is therefore more easily detected. Nonetheless, such a finding suggests that the surprise Recognition task was conceptually functional in capturing the effects of the self-/other-assignment manipulation in the Simon task—it successfully measured the degree to which participants encoded and memorized self-/other-referencing stimuli differently, in line with predictions from the self-reference effect. This provides cautious confidence to interpret the results of the Recognition task.

A caveated interaction effect (due to conflicting frequentist and Bayesian statistics) regarding H2 emerged. Participants in the Individual condition were not significantly affected by Assignment (i.e. recognition accuracy for Self-assigned versus Not-assigned items was not significantly different), while participants in the Joint condition recognized Self-assigned items significantly more accurately than Partner-assigned items. Such a finding suggests that the presence of a partner resulted in increased focus on a participant's own stimuli, in comparison to when participants have no partner. Interestingly, the direction of this effect was contrary to our hypothesis H2, which predicted that participants in the Joint condition would remember the Partner-assigned items more accurately than the equivalent Not-assigned items in the Individual condition.

One potential explanation for this outcome is that participants in the Joint condition employed a mental division of labour. This possibility is supported by adjacent literature investigating task-sharing in Stroop-like tasks; for example, one study used the picture–word interference task, where participants were tasked with naming a picture whilst ignoring a distractor word that overlayed the target picture [69]. This typically results in a semantic interference effect, where naming RT is increased when the target and distractor are semantically related (e.g. ‘dog’ and ‘cat’) in comparison to when the words are semantically unrelated (e.g. ‘dog’ and ‘cup’) [70]. In the adapted joint version of this task, participants first undertook the task individually, then alongside an alleged co-actor [69]. This alleged co-actor (who participants believed sat in another unspecified room, sharing the task with them) was tasked with either responding to the same picture stimuli target as the participant, or responding to the text-based distractor. Critically, the authors reported that the semantic interference effect was attenuated when the alleged co-actor was ‘in charge’ of the distractor (different-task condition), but not when the co-actor shared the same task as the participant (same-task condition). The authors concluded that participants in the different-task condition believed that their co-actor was responsible for the task-irrelevant distractor; this enabled the participants to filter out task-irrelevant information, resulting in the suppression of the distractor's interference effect. Similarly, it is possible that participants in the Joint conditions of our study recognized their partner as being ‘in charge’ of the partner-assigned items. Participants may have therefore passed the responsibility of processing and encoding that task-irrelevant information to their partner while focusing more on their self-assigned items in an unspoken division of labour.

However, an alternative explanation for increased focus on self-assigned stimuli in the Joint condition is that the presence of a partner introduced a sense of competition and an accompanying elevation in the participant's motivation for improving one's own performance. Past studies have demonstrated that inducing competition (in contrast to cooperation) using Tetris [25] or the Eriksen flanker task [71] prior to a Joint Simon task can attenuate the JSE (cf. [72], which reported that competition increased the magnitude of the JSE, similarly to cooperation). In the present study, this sense of competition between participants may have been unintentionally amplified due to the slight ‘gamification’ of the Simon task instructions; although competition was not overtly alluded to, participants were instructed to ‘catch’ the animal silhouettes of their own colour.^1^ Anecdotally, during the debriefing sessions at the end of the study, several participants in the Joint condition remarked that the tasks were quite enjoyable and game-like and that they tried to compare their performance in both the Simon task and the Recognition task with their partner's. The effects of this potentially heightened competitive mindset may have increased participants' attention towards their own stimuli during the Simon task [43], attenuating the JSE. Furthermore, in our study, this may have contributed to the enhanced encoding of the Self-assigned stimuli, which was captured in the subsequent Recognition task.

More generally, our results suggest that the presence of a partner during this task does in fact influence the degree of attention paid to self-assigned versus other-assigned stimuli. However, whether the underlying driver of this attentional difference is due to specific social mechanisms will require disambiguation through further investigations. Presently, the robustly null evidence for the effects of interpersonal closeness both on the Simon effect (H3a) and on Recognition task accuracy (H3b) may point more towards a low-level explanation, driven by attention biases towards self-relevant stimuli. Regarding the null findings towards H3a, our results differed from prior studies, which reported that interpersonal closeness (also measured using the IOS scale) positively correlated with the magnitude of the JSE [26]. However, due to the lack of a JSE in our Simon task and conflicting inferential statistics, we must exercise caution regarding the validity of these results and interpretations.

Hypothesis H4 asked whether the magnitude of SCEs in the Simon task would positively predict participants’ recognition accuracy for partner-assigned stimuli. Our results point to a robust null interaction effect, suggesting that variation in the degree to which participants were influenced by their partner (manifesting as differences in SCE magnitude) did not significantly impact participants' memory of partner-assigned stimuli. This result is unsurprising, considering the low variation in Simon task RTs across conditions. Therefore, we cannot necessarily conclude that such an effect does not exist—instead, it may be an artefact from the absence of the JSE in the present paradigm.

Crucial to considering the absence of a JSE effect, the present study's methodology deviated slightly from typical Joint Simon task designs. Firstly, instead of the typically used geometric shapes, we used images of animal silhouettes; secondly, the factor of Task Condition was tested between participants (whereas prior studies tested Condition as a repeated, within-participant factor; e.g. [26,61]); thirdly, stimuli presentation durations were standardized to 300 ms during the Simon task, regardless of how quickly participants responded.

The first modification of using animal silhouettes instead of geometric shapes may have increased the visual complexity of the stimuli. It is possible that this increased stimulus complexity could have heightened cognitive processing demands, therefore introducing interference and attenuation of the low-level Simon effect. However, previous studies have elicited the Simon effect even when using more visually complex stimuli (e.g. cartoon images of butterflies and frogs) [73,74]. Furthermore, because the key response criterion to the present Simon task was stimulus colour (and not stimulus shape), we posit that the impact of this modification was limited.

The second alteration—shifting from a within-participant to between-participant design—precluded the comparison of RT and accuracy performances within the same participant across Individual and Joint conditions. This alteration was necessary to accommodate the novel surprise Recognition task and potential impacts on statistical power were accounted for in our prior power analysis. As an unplanned analysis, we also tested whether the absence of the JSE was due to differences in analysis procedures: our originally planned analyses consisted of trial-level linear mixed-effects models, while the majority of similar studies used participant-level ANOVAs (e.g. [26,43]). However, for the present dataset, an ANOVA still did not reveal a JSE. We therefore reasonably conclude that the presently obtained null findings were due to methodological, rather than statistical, factors.

Finally, the standardization of stimuli presentation duration may have potentially reduced participants' sense of agency. For example, studies have reported that participants who felt like their actions do not have an observable effect—due to a >300 ms temporal lag between the participant's action and a perceptual event outcome—resulted in increased reaction time to their task in comparison to a no-temporal-lag condition [75,76]. The authors posited that this effect could be attributed to a decreased sense of agency and motivation to complete the ‘unrewarding’ task. However, our present paradigm resulted in mean RTs of approximately 340 ms. This indicates that most participants responded after the 300 ms stimuli presentation duration had elapsed (i.e. after the stimuli has disappeared), and therefore likely did not suffer much from an action–outcome disconnect, nor from a decrease in agency/motivation. Additionally, prior go/no-go (i.e. Individual and Joint) versions of the Simon task reported similar mean RTs of approximately 320–370 ms (e.g. [26,61,77]), suggesting that our present paradigm did not dramatically impact the general timescale of participants' responses.

The observed results call into question the general robustness of the Joint Simon task/effect to probing via experimental adaptations. For example, a recent study adapted the discrete pushbutton response of a typical Joint Simon task into a mouse-tracking response [78]. Akin to our findings, the authors did not replicate the key JSE between the Individual and Joint task conditions, although an SCE in the Standard Simon task was reported. The authors argued that the JSE may not generalize to more naturalistic response modalities, and that co-representation during the Joint Simon task may not be as pervasive as previously assumed. It is also possible that our non-replication indicates that the JSE is fairly fragile, in that its elicitation necessitates a specific suite of psychological and methodological contextual factors (e.g. graphically simple stimuli with minimal fluctuations between trials, binary response modalities). For example, under the referential coding account [32], systematic fluctuations in the stimuli between trials may increase the perceived differences in the self-/other-generated event codes, thereby decreasing the relative saliency of the left/right dichotomy and reducing stimulus–response spatial overlap. Nonetheless, we call for further research testing the boundaries of co-representation, both with regards to the JSE and in general, as a worthwhile endeavour; there is still a need to reconcile the causes of JSE non-replications with the wealth of positive results in the literature. Additionally, further replications using our present paradigm will be required to ascertain the robustness of our results.

Since we can only be confident that co-representation (or at least the behavioural indices of co-representation) is present when the conditions for the JSE are met, if a future study using a similar methodology to our present study is able to elicit the JSE, it would be insightful to compare the trends in the surprise Recognition task results. Should the hypothesized results (i.e. in H2) emerge, it may cement the notion that co-representation exists during the Joint Simon task when (and only when) the prerequisite conditions for triggering the JSE are present. This conclusion would suggest that participants do not co-represent their partner's stimuli when the conditions for the JSE are not met. One possible avenue to improve the present paradigm's likelihood of eliciting the JSE is to manipulate the degree of competition/cooperation between participants. There is evidence to suggest that an increased sense of cooperation between participants may increase the magnitude of the JSE [25,43,72]. However, there exists conflicting evidence regarding the effects of competition on self/other integration during task-sharing: some studies suggest that competition attenuates the JSE [25,43,71], whilst others found that competition increased the JSE, similar to cooperation [72]. An additional manipulation of cooperation/competition in future studies would assist in clarifying these inconsistent findings. Furthermore, such a line of future research may prove to be fruitful for informing the methodological boundary conditions of the Joint Simon paradigm, and the potential effects of social manipulations on self-/other-assigned stimuli memory patterns in the Recognition task.

To conclude, in using the adapted Joint Simon task to investigate the contents of co-representation, our results pointed towards a robust null JSE. Follow-up analyses of the novel Recognition task tentatively suggested that, in contrast to our hypothesized results, participants in the Joint condition tended to recall their own (self-assigned) stimuli even more than the equivalent (not-assigned) stimuli in the Individual condition. On the one hand, the non-replication of the JSE and the unexpected results in the Recognition task may have been due to methodological influences (e.g. unaccounted increase in the sense of competition between participants in the Joint condition). On the other hand, these null findings may reflect the relatively fragile nature of the JSE, insofar that the effect can be attenuated as a result of reasonably small methodological alterations.

## Data Availability

All experimental materials, questionnaires, data, and analysis scripts have been made available on the Open Science Framework: https://osf.io/mus39.

## Footnotes

^1^
Part of the experimental design decision-making process is informed by the potential follow-up with developmental samples. In the interest of testing the feasibility of applying this paradigm to child participants in future, the instructions were gamified to encourage interactivity and increase attention. The number of trials and study duration will also be limited for this reason.
